# Rational Constructed Ultra-Small Iron Oxide Nanoprobes Manifesting High Performance for *T*_1_-Weighted Magnetic Resonance Imaging of Glioblastoma

**DOI:** 10.3390/nano11102601

**Published:** 2021-10-02

**Authors:** Xiangyan Wang, Lei Chen, Jianxian Ge, Mohammad Javad Afshari, Lei Yang, Qingqing Miao, Ruixue Duan, Jiabin Cui, Chunyi Liu, Jianfeng Zeng, Jian Zhong, Mingyuan Gao

**Affiliations:** 1 College of Food Science & Technology, Shanghai Ocean University, Shanghai 201306, China; wxyixqj@163.com (X.W.); keirayang18@163.com (L.Y.); jzhong@shou.edu.cn (J.Z.); 2 Center for Molecular Imaging and Nuclear Medicine, State Key Laboratory of Radiation Medicine and Protection, School for Radiological and Interdisciplinary Sciences (RAD-X), Soochow University, Suzhou 215123, China; chenlei199303@gmail.com (L.C.); jxge@stu.suda.edu.cn (J.G.); moja.afshari@gmail.com (M.J.A.); qqmiao@suda.edu.cn (Q.M.); rxduan@suda.edu.cn (R.D.); jiabin.cui@suda.edu.cn (J.C.); cyliu19@suda.edu.cn (C.L.); 3 Collaborative Innovation Center of Radiological Medicine of Jiangsu Higher Education Institutions, Soochow University, Suzhou 215123, China

**Keywords:** ultra-small iron oxide nanoparticles, glioblastoma, MRI, *T*_1_ contrast agent

## Abstract

Precise diagnosis and monitoring of cancer depend on the development of advanced technologies for in vivo imaging. Owing to the merits of outstanding spatial resolution and excellent soft-tissue contrast, the application of magnetic resonance imaging (MRI) in biomedicine is of great importance. Herein, Angiopep-2 (ANG), which can simultaneously help to cross the blood-brain barrier and target the glioblastoma cells, was rationally combined with the 3.3 nm-sized ultra-small iron oxide (Fe_3_O_4_) to construct high-performance MRI nanoprobes (Fe_3_O_4_-ANG NPs) for glioblastoma diagnosis. The in vitro experiments show that the resultant Fe_3_O_4_-ANG NPs not only exhibit favorable relaxation properties and colloidal stability, but also have low toxicity and high specificity to glioblastoma cells, which provide critical prerequisites for the in vivo tumor imaging. Furthermore, in vivo imaging results show that the Fe_3_O_4_-ANG NPs exhibit good targeting ability toward subcutaneous and orthotopic glioblastoma model, manifesting an obvious contrast enhancement effect on the *T*_1_-weighted MR image, which demonstrates promising potential in clinical application.

## 1. Introduction

Magnetic resonance imaging (MRI) has been demonstrated as a powerful technique for the visualization of the anatomical structure with high spatial resolution [1,2,3]. Compared with computed tomography (CT) [4], positron emission tomography (PET), and single photon emission computed tomography (SPECT) [5], MRI offers several advantages including excellent soft-tissue contrast, the absence of potentially destructive ionizing radiation, and multi-functional imaging with diverse applications [6,7,8].

During MRI scanning, proton nuclei would return to the equilibrium state after removing the resonant radio frequency pulses. This process results in two types of relaxation processes, namely longitudinal relaxation and transverse relaxation, which is defined as *T*_1_ and *T*_2_ relaxation, respectively [3]. Generally, the MRI contrast agents, which are responsible for accelerating the relaxation of protons in water molecules and subsequently providing better contrast for the region of interest, can be divided into two categories [9,10]. The *T*_1_ contrast agent causes positive/brighter contrast enhancement, while the *T*_2_ contrast agent causes negative/dark contrast enhancement [8,10]. Currently, the commonly used clinical products are gadolinium chelate-based *T*_1_ contrast agents [11]. Although the USA Food and Drug Administration (FDA) has approved several different types of Gd-based agents for clinical diagnoses, a number of undesirable side effects such as nephrogenic systemic fibrosis which arise from the leaching of Gd3+, has caused serious concerns about their usage for in vivo applications [12,13]. Iron oxide nanoparticles are alternative MRI contrast agents that were developed as *T*_2_ contrast agents for more than 30 years due to their excellent magnetic properties [9,14], good biocompatibility [15], biodegradability [16], and low toxicity [17]. However, the *T*_2_-weighted signals can usually be confused by bleeding, calcification, metal deposits, and other susceptibility artifacts, limiting their further clinical applications [18,19,20]. Nevertheless, owing to the surface canting effect, ultra-small iron oxide nanoparticles (<5 nm) are shown to be able to enhance *T*_1_ contrast considerably [15,21], opening a promising avenue for precise cancer diagnosis through in vivo imaging.

Glioblastoma multiforme (GBM), which is one of the most malignant, life-threatening, and aggressive tumors of the central nervous system (CNS), can put the patient to death in an average 14-month period after diagnosis with a low 5-year relative survival rate of less than 5% [22,23,24,25]. Since no effective and non-operative treatment has been suggested to date, detecting the tumor boundaries with high precision is of great importance for surgically removing the tumor cells. However, the delivery and diffusion of medicine or contrast agents to the CNS are severely restricted by the highly sensitive blood-brain barrier (BBB), posing serious challenges to effectively targeting the tumor for diagnostic or therapeutic purposes [26,27,28]. To address this problem, several strategies including the adsorption mediated transcytosis (AMT) [29], carrier-mediated transport (CMT) [30], and receptor-mediated transcytosis (RMT) [31] were developed. Among the above-mentioned methods, RMT is widely reported due to the specific binding and strong affinity between the carrier and receptor, which provides a potential solution for the efficient visualization and diagnosis of GBM [32,33]. As was reported, the low-density lipoprotein receptor-related protein 1 (LRP1) is overexpressed in both the brain capillary endothelial cells (BCECs) of BBB and glioblastoma cells and exhibits specific affinity with Angiopep-2 (ANG), which was demonstrated with high efficiency for the crossing of BBB [28,31,32,34,35]. Thus, the combination of Angiopep-2 with ultra-small iron oxide nanoparticles might be able to serve as an effective contrast agent for the visualization of GBM with high precision.

**Scheme 1:**
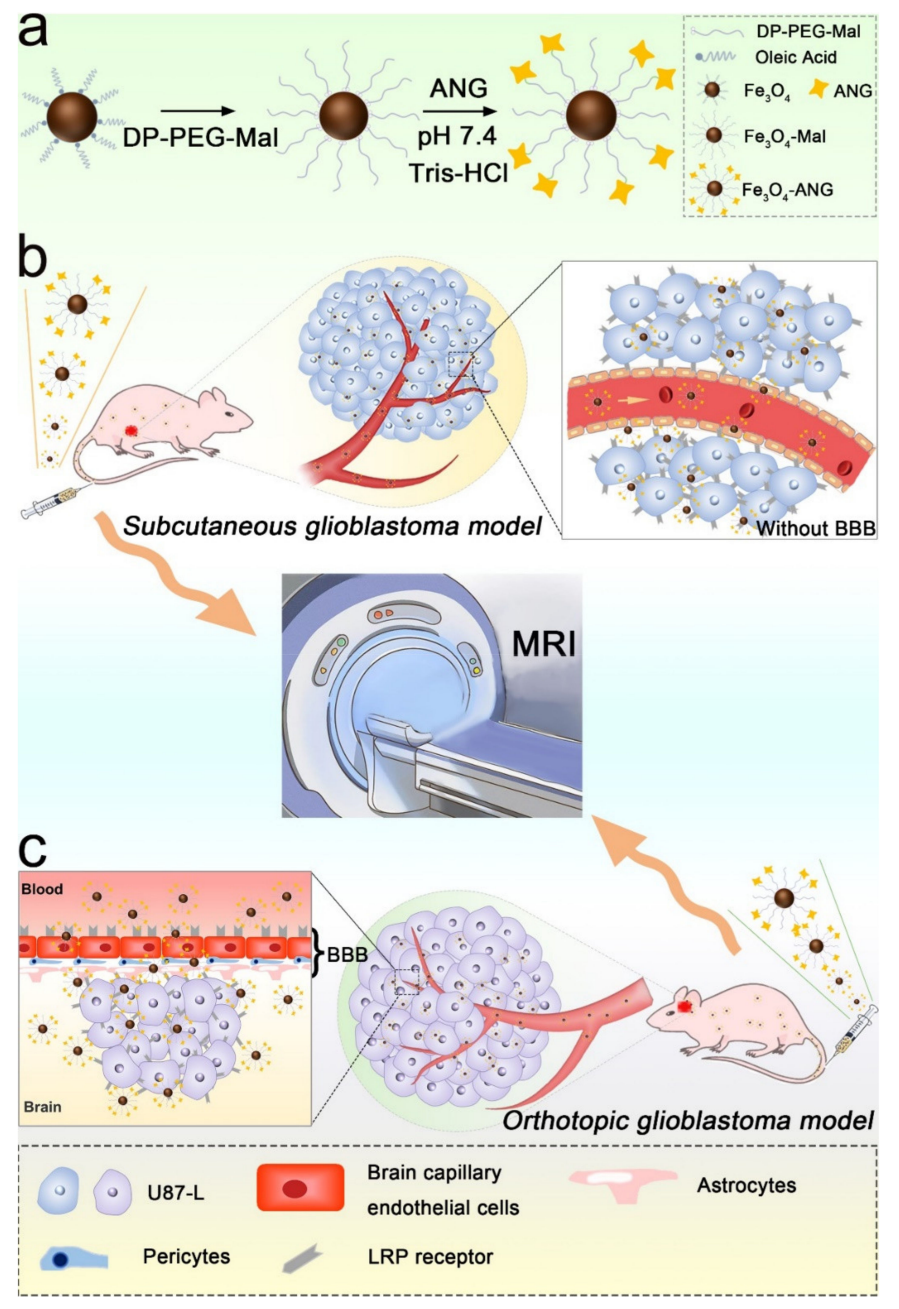
(**a**) The strategy for the design of Fe_3_O_4_-Mal and Fe_3_O_4_-ANG nanoprobe. (**b**) Fe_3_O_4_-Mal or Fe_3_O_4_-ANG NPs was ingested by the subcutaneous glioblastoma in the absence of BBB. (**c**) Fe_3_O_4_-Mal or Fe_3_O_4_-ANG NPs target the orthotopic glioblastoma with LRP1 receptor for the cross through of BBB.

The main purpose of this study was to construct Angiopep-2-functionalized ultra-small iron oxide nanoprobes (Fe_3_O_4_-ANG NPs), evaluating their potential capabilities to be used as contrast agents for the diagnosis of GBM. As shown in Scheme 1, the iron oxide nanoparticles were first modified with the maleimide-ended polyethylene glycol (PEG), and then conjugated with thiol-functionalized Angiopep-2 through a facile click reaction to obtain the MRI nanoprobes. The physiochemical properties, biocompatibility, and targeting specificity of the resultant nanoprobes were fully investigated. In addition, the in vivo MRI imaging performances were evaluated on both subcutaneous and orthotopic glioblastoma mice models. All the experimental results suggest that the proposed Fe_3_O_4_-ANG NPs might have great potential in clinical applications.

## 2. Materials and Methods

### 2.1. Materials

Iron (III) acetylacetonate (Fe(acac)3, 98%), oleic acid (OA, 85%), oleylamine (OAm, 80%), isopropyl alcohol (99%), cyclohexane (99.5%), and tris(2-carboxyethyl) phosphine (TCEP, 98%) were purchased from Aladdin Biochemical Technology Co., Ltd. (Shanghai, China). Tetrahydrofuran (THF, 99.5%) and acetone (99.5%) were purchased from Sinopharm Chemical Reagent Co., Ltd. (Shanghai, China). Angiopep-2 (TFFYGGSRGKRNNFKTEEYC, molecular weight 2.4 kDa) was purchased from Xi’an Ruixi Biological Technology Co., Ltd. (Xi’an, China). Polyethylene glycol (molecular weight 2.0 kDa) bearing a maleimide group at one end and a diphosphate group at the other end, denoted as DP-PEG_2000_-Mal, was purchased from Suzhou Xinying Bio-Medical Technology Co., Ltd. (Suzhou, China). Fetal bovine serum (FBS, Hyclone), phosphate buffered saline (PBS), Dulbecco’s Modified Eagle Medium (DMEM), Roswell Park Memorial Institute (RPMI-1640), penicillin–streptomycin and 0.25% (*w*/*v*) trypsin-EDTA solution were purchased from Solarbio Science & Technology Co., Ltd. (Beijing, China). CCK-8 was purchased from APExBIO Co., Ltd. (Houston, TX, USA). Prussian blue stain and formalin were purchased from Shanghai Yuanye Biotechnology Co., Ltd. (Shanghai, China). All reagents were used without any further purification.

### 2.2. Cells and Animals

Human glioblastoma cells (U87-L) were purchased from Nanjing Kebai Biotechnology Co., Ltd. (Nanjing, China). The murine brain capillary endothelial cells (BCECs) were obtained from Beijing Beina Chuanglian Institute of Biotechnology (Beijing, China). U87-L were routinely cultured in DMEM supplemented with 10% (FBS) and 1% penicillin/streptomycin. BCECs were cultured in RPMI-1640 supplemented with 10% FBS and 1% penicillin/streptomycin. The incubator was maintained at 37 °C, 5% CO_2_ and 95% humidity. Specific pathogen free (SPF) female nude mouse (4–5 weeks old) was purchased from Changzhou Cavins Laboratory Animal Co., Ltd. (Changzhou, China). They were maintained under standard conditions for a week before the experiment. All animal experiments reported herein were carried out according to the protocols approved by Soochow University Laboratory Animal Center.

### 2.3. Characterization

Transmission electron microscope (TEM) images were captured with an Tecnai G20 (FEI, USA) transmission electron microscope operating at an acceleration voltage of 200 kV to determine the size and morphology of nanoparticles. The hydrodynamic size and zeta potential were measured by dynamic light scattering (DLS) using a Zetasizer Nano ZS90 (Malvern, Britain) at 25 °C. The relaxation rate of nanoparticles was obtained using a preclinical MRI scanner (MRS 3000, MR Solution, Britain).

### 2.4. Establishment of Glioblastoma Models

To establish the subcutaneous glioblastoma model, the U87-L cells in the logarithmic phase were treated with 0.25% trypsin-EDTA and diluted into cell suspension (1 × 10^7^ cell mL^−1^) with PBS. Then U87-L cells suspension (100 μL) was subcutaneously injected into the right posterior dorsal region of each female nude mouse (body weight of 16–18 g) to establish the subcutaneous glioblastoma model. By monitoring the tumor growth, mice with a tumor volume of ~100 mm^3^ were used for the following imaging studies. For the orthotopic glioblastoma model, U87-L cells were treated as described above and diluted into cell suspension (1 × 10^8^ cell mL^−1^) with PBS. After the mouse is anesthetized, the head skin was incised by a sterile scalpel and U87-L cells (5 μL) were then injected into the striatum. The injection location was determined with the assistance of brain stereotaxic apparatus. After that, the preclinical MRI scanner was used to perform *T*_2_-weighted imaging on tumor-bearing mice to observe the tumor growth.

### 2.5. Synthesis, Purification and Characterization of Fe_3_O_4_ NPs

In the current study, the hydrophobic ultra-small iron oxide nanoparticles (Fe_3_O_4_ NPs) were first prepared in a flow synthesis device equipped with a tubular reactor (inner diameter 8 mm) [36]. Typically, 8.33 g (23.6 mmol) of the iron (III) acetylacetonate, 40.1 g (142 mmol) of oleic acid, and 38.0 g (142 mmol) of oleylamine were dissolved in 1 L of isopropyl alcohol to prepare a flowing reaction liquid, which was pumped into the tubular reactor by a high-pressure constant-current pump, and the flow rate, reaction temperature, and pressure were set to 200 mL min^−1^, 270 °C, and 5.1 MPa, respectively. After the reaction, the solution was cooled by water and the crude product solution was collected in a sample bottle, which was then washed with acetone/cyclohexane two times. The final product was dissolved in cyclohexane and stored at 4 °C.

### 2.6. Ligand Exchange with DP-PEG_2000_-Mal to Prepare Fe_3_O_4_-Mal NPs

In order to obtain Fe_3_O_4_-Mal NPs, which are modified with DP-PEG_2000_-Mal, 100 mg of DP-PEG_2000_-Mal were dissolved in 3 mL of THF containing 10 mg hydrophobic Fe_3_O_4_ NPs [37]. Then the reaction mixture was subjected to ultrasonication for 2 h at 40 °C. Subsequently, the Fe_3_O_4_ NPs were precipitated and washed by cyclohexane and then dried under vacuum at room temperature. The final product was dissolved in pure water and further purified by ultrafiltration using 30 kDa MWCO centrifugal filter.

### 2.7. Conjugation of ANG to Fe_3_O_4_-Mal Surface to Prepare Fe_3_O_4_-ANG NPs

The modification of ANG on the surface of Fe_3_O_4_-Mal NPs was based on the fact that the maleimide group can specifically react with thiol groups in a neutral environment to form stable sulfide bonds. Typically, Fe_3_O_4_-Mal NPs (containing 2 mg Fe) were reacted with ANG (1.45 mg) in Tris-HCl (pH 7.4) for 12 h at room temperature. The obtained Fe_3_O_4_-ANG NPs were purified for four times by ultrafiltration using 30 kDa MWCO centrifugal filter and stored at 4 °C for later use. In addition, the absorbance spectrum of the filtrate was measured with a UV spectrophotometer at 275 nm to determine the non-reacted ANG.

### 2.8. Measurement of the Relaxivity of Fe_3_O_4_-Mal and Fe_3_O_4_-ANG NPs by MRI

Fe_3_O_4_-Mal, Fe_3_O_4_-ANG NPs and Gd-DTPA were diluted to 0 mM, 0.05 mM, 0.1 mM, 0.2 mM, 0.3 mM, 0.4 mM, 0.5 mM with MilliQ water. Then, they were loaded into the Duchenne tubule and scanned with a small animal MRI system of 3T. The parameters for *T*_1_ measurements were set as follows: echo time (TE) = 11 ms, repetition time (TR) = 200~5000 ms, field of view (FOV) = 40 × 40 mm^2^, slice thickness = 2 mm.

### 2.9. Hydrodynamic Size, Zeta Potential and Stability of Fe_3_O_4_-Mal and Fe_3_O_4_-ANG NPs

Fe_3_O_4_-Mal and Fe_3_O_4_-ANG NPs were diluted to 50 μg mL^−1^ with HEPES buffer (pH 7.4). Then the hydrodynamic size and zeta potential of the two kinds of NPs were determined by a Zetasizer Nano ZS90 (Malvern, Britain). To monitor the colloidal stability, the Fe_3_O_4_-Mal and Fe_3_O_4_-ANG NPs were diluted with PBS to 50 μg mL^−1^. Then the hydrodynamic size and zeta potential of the two kinds of NPs were measured at different time points. 

### 2.10. Cytotoxicity Assessment In Vitro

Herein, standard CCK-8 assays were adopted to evaluate the cytotoxicity of the resultant nanoprobes. Firstly, U87-L and BCECs cells in the logarithmic growth phase were treated with 0.25% trypsin-EDTA solution and seeded into a 96-well plate at a density of 8 × 10^3^ cells well-1. These cells were incubated overnight at 37 °C, 5% CO_2_ and 95% humidity to ensure the cell attach. Then, the culture medium was replaced with a fresh medium containing Fe_3_O_4_-Mal or Fe_3_O_4_-ANG NPs with different Fe concentrations (0, 50, 100, 200, 400, 600 μg mL^−1^) and incubated in the same environment for another 24 h. Finally, the medium was removed and washed by PBS, then 100 μL culture medium containing 10 μL CCK-8 solutions was added to each well. The absorbance at 450 nm of each well was determined after the incubation of 1–4 h. The cell viability can be obtained by the following formula, A = (As − Ab)/(Ac − Ab) (where As, Ac and Ab are the absorbance values of the test, control and blank wells, respectively).

### 2.11. Prussian Blue Staining of Fe_3_O_4_-Mal and Fe_3_O_4_-ANG NPs In Vitro

Prussian blue staining was used to evaluate the uptake of Fe_3_O_4_-Mal and Fe_3_O_4_-ANG NPs by U87-L cells. Approximately 3 × 10^4^ U87-L cells were seeded in 24-well plate and incubated overnight at 37 °C under 5% CO_2_ and 95% humidity to allow a firm adherence. Then, a fresh medium containing Fe_3_O_4_-Mal or Fe_3_O_4_-ANG NPs with different Fe concentrations (0, 100 μg mL^−1^) were introduced after removal of the old medium. After being incubated for 24 h, the Fe-containing medium was removed and the wells were washed three times with PBS and then fixed with 4% paraformaldehyde for 30 min at room temperature. After discarding the 4% paraformaldehyde and washing the wells three times by PBS, these cells were treated with pearls reagent (4% potassium ferrocyanide and 12% HCl, volume ratio 1:1) for 1 h at room temperature. The staining was observed by an inverted biological microscope.

### 2.12. In Vivo MRI Assessment

To evaluate the contrast enhancement effect of Fe_3_O_4_-Mal and Fe_3_O_4_-ANG NPs in vivo, both subcutaneous and orthotopic glioblastoma mice model were used. After being anesthetized, the tumor-bearing mice were intravenously injected with the nanoprobes (0.1 mmol Fe kg^−1^ bodyweight). The *T*_1_-weighted images were acquired by the preclinical MRI scanner at specific time points including pre- and post-injection. The scan parameters are as follows: echo time (TE) = 11 ms, repetition time (TR) = 850 ms, field of view (FOV) = 40 × 40 mm^2^, slice thickness = 1 mm. After the MRI scans, the mice were sacrificed by euthanasia and the tumor was dissected and fixed in 10% formalin for further H&E staining and Prussian blue staining.

## 3. Results and Discussion

### 3.1. Synthesis and Characterization of Fe_3_O_4_, Fe_3_O_4_-Mal and Fe_3_O_4_-ANG NPs

**Figure 1:**
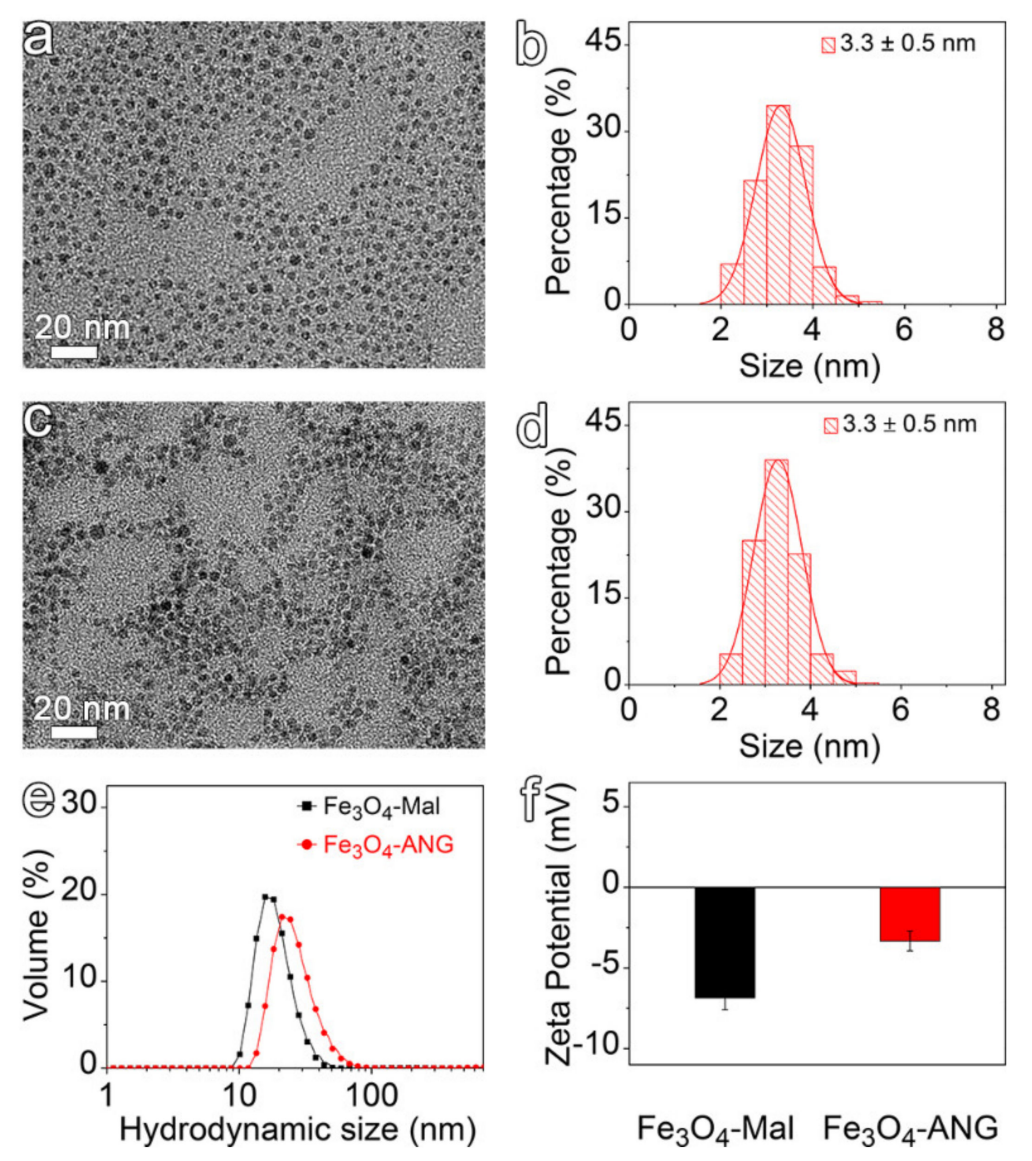
(**a**) TEM analysis and (**b**) size distribution of as-synthesized hydrophobic Fe_3_O_4_ NPs. (**c**) TEM image and (**d**) size distribution of Fe_3_O_4_-ANG NPs. (**e**) Hydrodynamic size distribution profiles and (**f**) Zeta potential values of Fe_3_O_4_-Mal and Fe_3_O_4_-ANG NPs.

The hydrophobic Fe_3_O_4_ NPs were prepared by the flow synthesis method reported previously with slight modification [36]. The transmission electron microscopy (TEM) image of the as-synthesized nanoparticles and their corresponding particle size distribution are shown in Figure 1a,b, respectively, confirming that the nanoparticles exhibit a spherical shape with an average particle size of 3.3 ± 0.5 nm. To render the ultra-small sized Fe_3_O_4_ NPs water-solubility and functionalization ability, the oleate ligands on the particle surface were replaced by DP-PEG_2000_-Mal using a common ligand exchange strategy, thus obtaining the Fe_3_O_4_-Mal NPs (Figure S1). Through the maleimide residues on the Fe_3_O_4_-Mal NPs surface, thiol-functionalized ANG was further covalently conjugated onto the particle surface by the click reaction. To remove the unreacted ANG, the reaction mixture was further purified by ultrafiltration to obtain the Fe_3_O_4_-ANG NPs. The TEM results indicate that there are no significant changes in the morphology and the particle size distribution after the surface modification (Figure 1c,d). However, the DLS results of Fe_3_O_4_-Mal and Fe_3_O_4_-ANG NPs shown in Figure 1e reveal that the hydrodynamic size is slightly increased after the conjugation of ANG, which can be explained by the high molecular weight of the ANG that attached to the particle surface [37]. The polydispersity index (PDI) of the nanoparticle has no significant change before and after the conjugation of ANG (0.33 vs. 0.27), indicating no aggregation of particles occurs during the conjugation process. In addition, the zeta potential also increased after the ANG modification (Figure 1f), which may be attributed to the positive charge of ANG. All of the above results indicate that ANG is successfully conjugated with the nanoparticle surface. The sufficient ANG surface modification is the most critical prerequisite for the high targeting capability of Fe_3_O_4_-ANG NPs. Therefore, in order to quantify the number of ANG molecules attached to the surface of each particle, the unreacted ANG concentration in the filtrate was determined by a UV spectrophotometer based on a standard curve established at 275 nm (Figure S2). The results show that the number of ANG polypeptides coupled on each nanoparticle is estimated to be 7.

### 3.2. Relaxation Performance and Stability of Fe_3_O_4_-Mal NPs, Fe_3_O_4_-ANG NPs and Gd-DTPA

**Figure 2:**
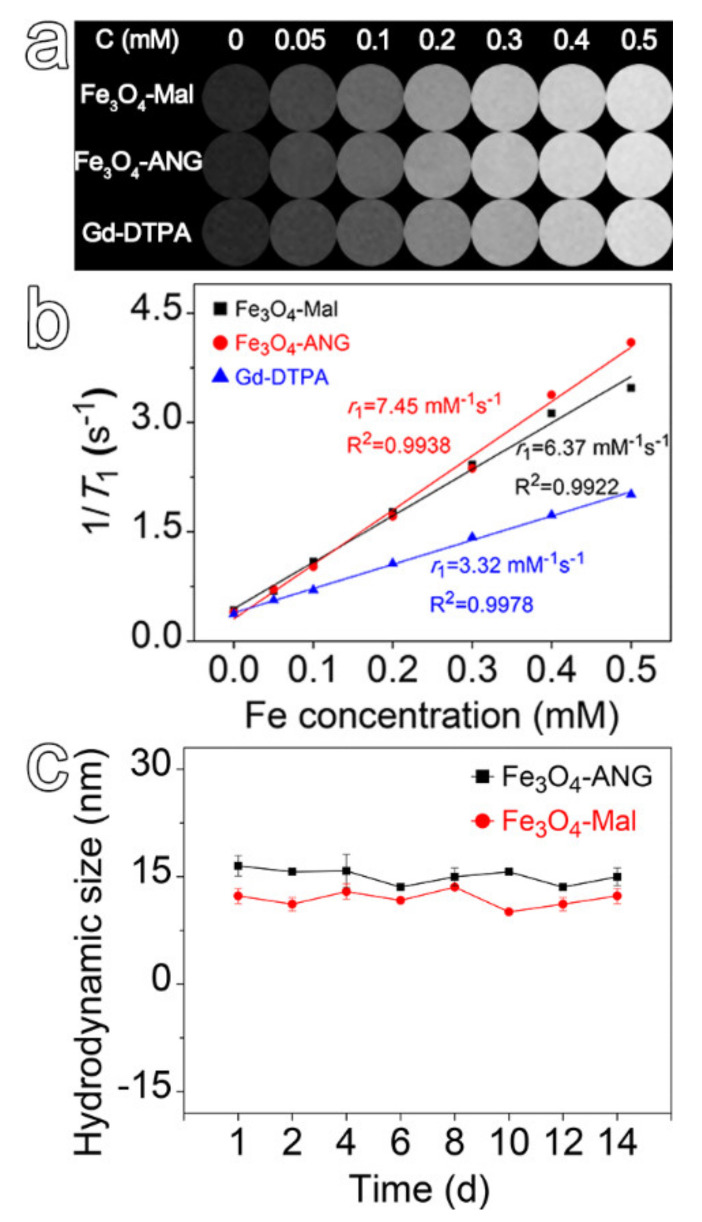
(**a**) *T*_1_-weighted images of the Fe_3_O_4_-Mal, Fe_3_O_4_-ANG NPs, and Gd-DTPA. (**b**) Linear regression fitting of the relaxivity of Fe_3_O_4_-Mal, Fe_3_O_4_-ANG NPs, and Gd-DTPA for extracting the longitudinal relaxivity *r*_1_. (**c**) Stability evaluation for Fe_3_O_4_-Mal and Fe_3_O_4_-ANG NPs incubated in PBS for 14 days.

The relaxation measurements were performed under 3 T to evaluate the performance of the resultant nanoprobes as MRI contrast agents. Figure 2a shows the *T*_1_-weighted MR images of Fe_3_O_4_-Mal NPs, Fe_3_O_4_-ANG NPs, and Gd-DTPA (Magnevist) at different concentrations. As it is evident in the figure, although increasing the concentration gives rise to a significant enhancement in signal intensity for all the evaluated contrast agents, Fe_3_O_4_-Mal and Fe_3_O_4_-ANG NPs present stronger *T*_1_ contrast effects compared to that of Gd-DTPA. Further linear regression fitting of the experimentally determined longitudinal relaxation rates (Figure 2b) shows that the molar relaxivity *r*_1_ of Fe_3_O_4_-Mal and Fe_3_O_4_-ANG NPs are 6.37 mM^−1^ s ^−1^ and 7.45 mM^−1^ s^−1^, respectively, which are much higher than that of 3.32 mM^−1^ s^−1^ for Gd-DTPA. In addition, the transverse relaxivity *r*_2_ of Fe_3_O_4_-Mal and Fe_3_O_4_-ANG NPs are 33.2 mM^−1^ s^−1^ and 36.6 mM^−1^ s^−1^ (Figure S3), respectively, giving rise to *r*_2_/*r*_1_ of 5.21 and 4.91. The high longitudinal relaxivity and low *r*_2_/*r*_1_ ratio indicate the resultant ultra-small iron oxide nanoprobes are promising *T*_1_ contrast agents for tumor diagnosis. To assess the colloidal stability of the resultant nanoprobes, the Fe_3_O_4_-Mal and Fe_3_O_4_-ANG NPs were incubated in PBS, and the hydrodynamic sizes were monitored by DLS. As shown in Figure 2c, the hydrodynamic sizes show negligible change within 14 days, indicating both the Fe_3_O_4_-Mal and Fe_3_O_4_-ANG NPs present excellent colloidal stability in PBS, which is a commonly used solution to mimic the physiological conditions inside the body. The above results support the fact that DP-PEG_2000_-Mal can effectively render the ultra-small Fe_3_O_4_ NPs satisfying water solubility and colloidal stability by suppressing the dipole–dipole interactions among Fe_3_O_4_ NPs, probably due to the steric hindrance effect of PEG. The highly colloidal stability built a solid foundation for further exploring in vivo applications of the resultant nanoprobes.

### 3.3. Cytotoxicity of the Fe_3_O_4_-Mal and Fe_3_O_4_-ANG NPs

**Figure 3:**
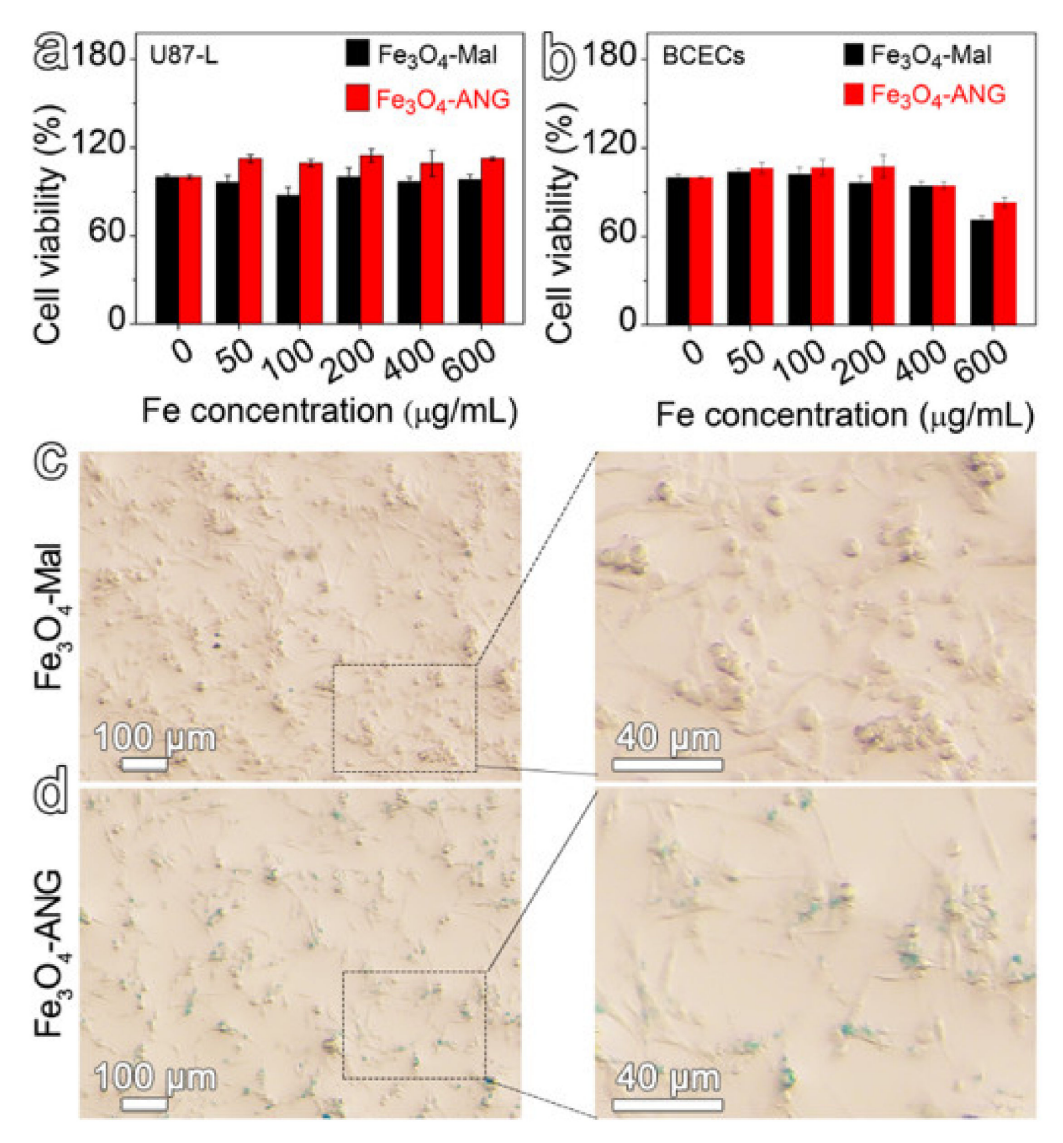
U87-L (**a**) and BCECs (**b**) cells viabilities by CCK-8 assay after incubating with varied concentrations of Fe_3_O_4_-Mal or Fe_3_O_4_-ANG NPs for 24 h. Micrographs of Prussian blue staining of U87-L cells incubated with Fe_3_O_4_-Mal NPs (**c**) or Fe_3_O_4_-ANG NPs (**d**) for 24 h, together the corresponding high magnification micrographs.

The cytotoxicity of Fe_3_O_4_-Mal and Fe_3_O_4_-ANG NPs was evaluated via the standard cell counting kit 8 (CCK-8) assays on the proliferation of U87-L and BCECs cells. As depicted in Figure 3a,b, both the Fe_3_O_4_-Mal and Fe_3_O_4_-ANG NPs exhibit low cytotoxicity within the studied concentration range. The cell viability remains higher than 90% even when the Fe concentration reaches 400 μg mL^−1^. The low cytotoxicity of the obtained ultra-small Fe_3_O_4_ nanoprobes is expected since the outstanding biocompatibility of iron oxide nanoparticles was demonstrated by a large number of studies.

### 3.4. In Vitro Specificity Studies of MRI Nanoprobes

To actively target the glioblastoma, the ANG polypeptide was conjugated to the Fe_3_O_4_-Mal NPs through a click reaction between the maleimide moiety of the PEG ligand on the particle surface and thiol group from the ANG, thus obtaining Fe_3_O_4_-ANG NPs. The previous results have demonstrated the successful coupling of ANG onto the surface of Fe_3_O_4_ nanoparticles. To further validate the target-binding specificity, the resultant nanoprobes were assessed through cell binding assays, in which the Fe_3_O_4_-Mal or Fe_3_O_4_-ANG NPs were co-incubation with U87-L cells. After being incubated for 24 h, the cells were washed and fixed, followed by Prussian blue staining to evaluate the cell uptake of nanoprobes. As shown in Figure 3c,d, the cells incubated with Fe_3_O_4_-ANG NPs present obvious blue staining, which is quite different from the Fe_3_O_4_-Mal group that shows no evident blue color. The results clearly reveal that the ANG on the particle surface can dramatically increase the binding affinity between U87-L cells and Fe_3_O_4_-ANG NPs.

### 3.5. In Vivo MR Imaging of the Glioblastoma

**Figure 4:**
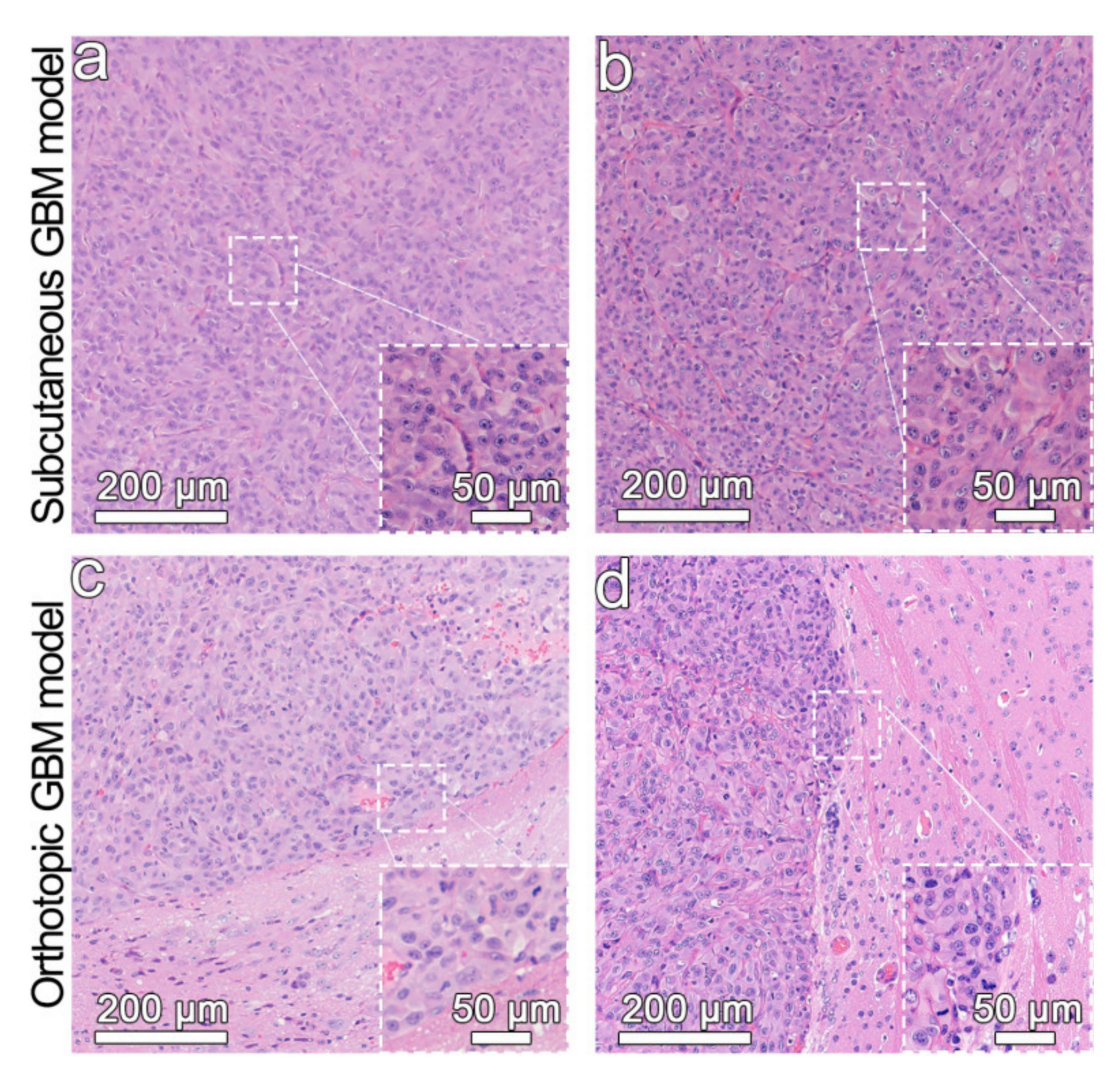
H&E staining of the subcutaneous glioblastoma model (**a**,**b**) and the orthotopic glioblastoma model (**c**,**d**). The inserts illustrate the high magnification micrographs.

Functional nanoparticles were widely used for tumor imaging by taking the advantages of the leaky vasculature of tumors, which is known as passive tumor targeting that attributes to the enhanced permeability and retention (EPR) effect. To further increase the tumor accumulation efficiency of nanoparticles, various types of targeting molecules were conjugated to the particle surface to thus enhance the binding affinity, leading to active targeting. In this work, ANG that can specifically bind with the LRP1 overexpressed in glioblastoma cells and brain capillary endothelial cells of BBB was adopted to realize the active tumor targeting imaging. The above results have demonstrated that the synthesized Fe_3_O_4_-ANG nanoprobes show excellent colloidal stability, outstanding biocompatibility, and high cell specificity. To further assess the in vivo tumor imaging ability of the resultant nanoprobes, both subcutaneous and orthotopic glioblastoma models were established. The orthotopic glioblastoma models can better reflect the nature of cancers, while the subcutaneous glioblastoma model offers an opportunity for comparing the imaging effect with/without BBB. As illustrated in Figure 4, the hematoxylin and eosin (H&E) staining images of both tumor models indicate that the cells were aggregated and emerged irregularly in shape, demonstrating the successful establishment of the glioblastoma model.

**Figure 5:**
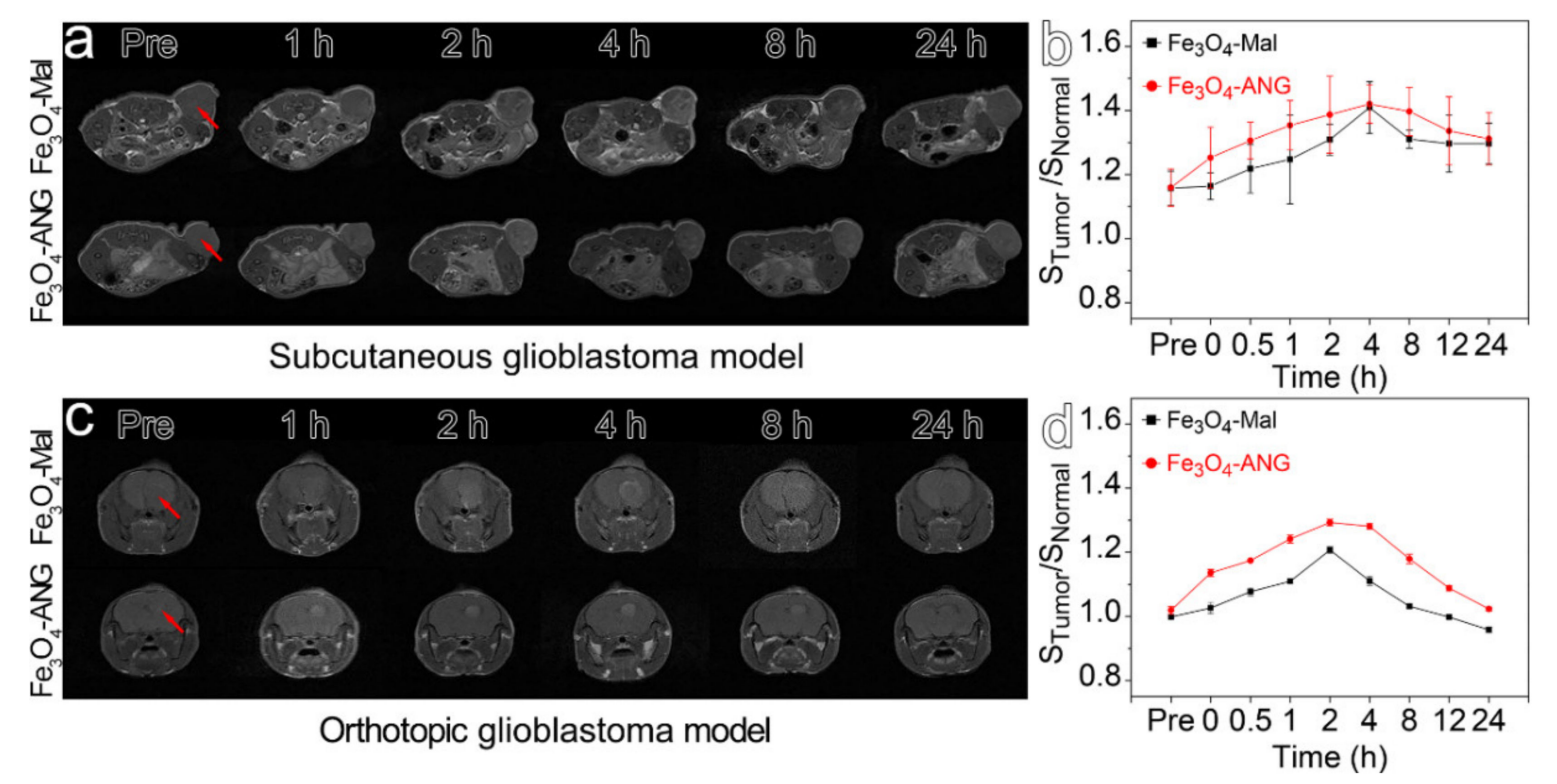
(**a**) MRI of the subcutaneous glioblastoma model within 24 h after injection of Fe_3_O_4_-Mal or Fe_3_O_4_-ANG NPs. (**b**) Tumor signal trends for the subcutaneous glioblastoma model after injection of Fe_3_O_4_-Mal or Fe_3_O_4_-ANG NPs. (**c**) MRI of the orthotopic glioblastoma model within 24 h after injection of Fe_3_O_4_-Mal or Fe_3_O_4_-ANG NPs. (**d**) Tumor signal trends for the orthotopic glioblastoma model after injection of Fe_3_O_4_-Mal or Fe_3_O_4_-ANG NPs.

Then, through the tail vein, the Fe_3_O_4_-Mal or Fe_3_O_4_-ANG NPs as mentioned above were injected into the tumor-bearing mice with an injection dose of 0.1 mmol kg^−1^. The *T*_1_-weighted images acquired with the 3 T MRI scanner before and at different time points post-injection (0.5 h, 1 h, 2 h, 4 h, 8 h, 12 h and 24 h) are provided in Figure 5. The MR contrast enhancements were gradually increased by both the Fe_3_O_4_-Mal and Fe_3_O_4_-ANG NPs and reached to its maximum at 4 h and 2 h with respect to subcutaneous and orthotopic glioblastoma models, respectively. The time difference for reaching the maximum contrast is probably caused by the differences between the microenvironments and blood supplies of the subcutaneous and orthotopic glioblastoma models. Although the Fe_3_O_4_-Mal and Fe_3_O_4_-ANG NPs present similar signal evolution tendencies for both kinds of tumor models, the Fe_3_O_4_-ANG NPs represent higher contrast enhancement than the Fe_3_O_4_-Mal NPs, which can be interpreted by the increased tumor uptake of the Fe_3_O_4_-ANG NPs through active targeting. In addition, the contrast difference between the Fe_3_O_4_-Mal and Fe_3_O_4_-ANG NPs for the orthotopic glioblastoma model is more significant than the subcutaneous one, indicating the ANG modification can effectively help nanoprobes to cross the BBB and accumulate in the tumor.

**Figure 6:**
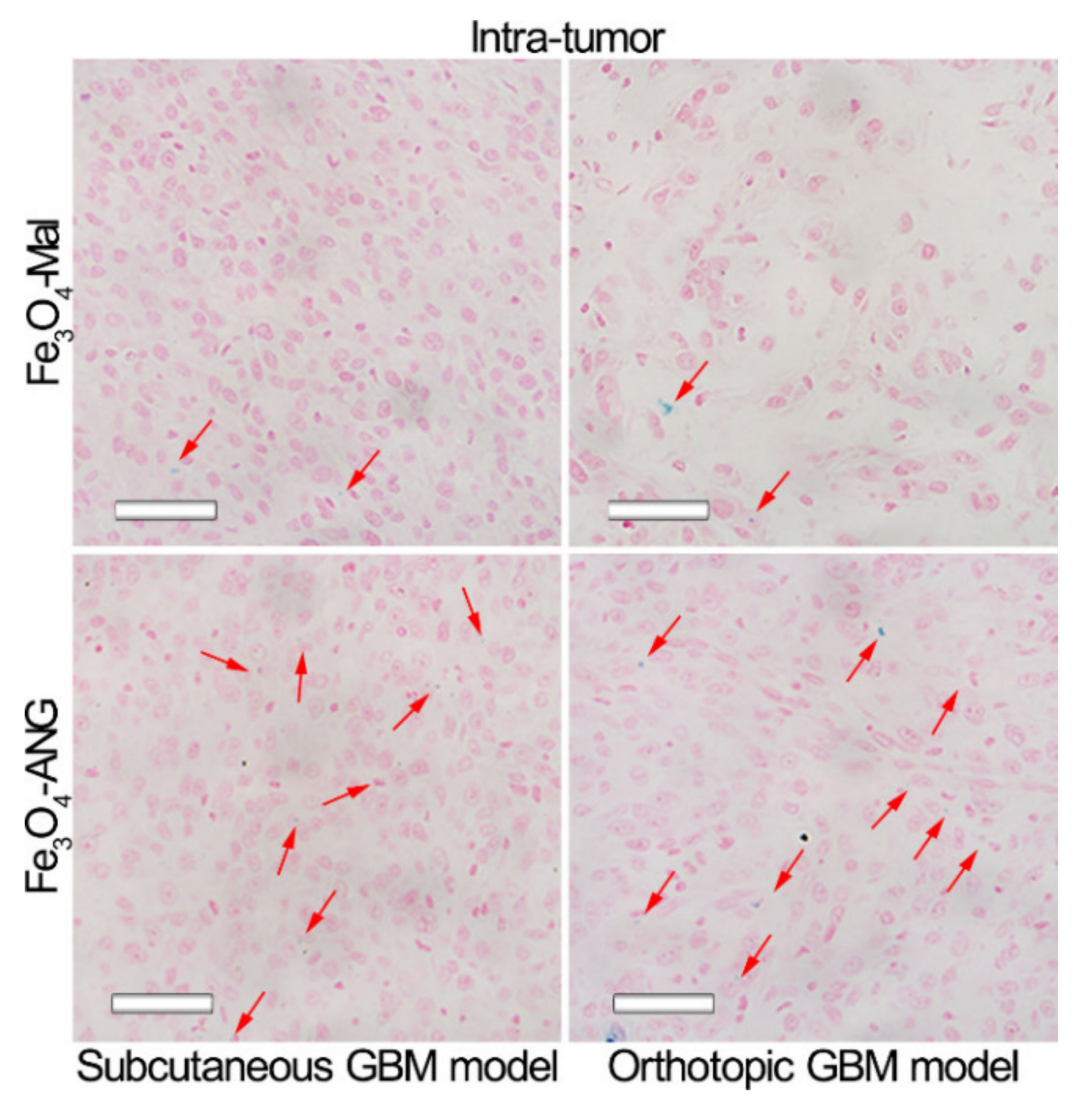
Prussian blue staining of the subcutaneous glioblastoma model and the orthotopic glioblastoma model after injection of Fe_3_O_4_-Mal or Fe_3_O_4_-ANG NPs. The Scale bar is 50 μm.

To further demonstrate the targeting imaging effects, MRI scans were performed on the parallel tumor-bearing mice. However, the scans were stopped at the time point with the maximum contrast enhancement effect, i.e., 4 h and 2 h for subcutaneous and orthotopic glioblastoma models, respectively. Then, the mice were sacrificed and the tumors were excised for Prussian blue staining. The obtained *T*_1_-weighted images (Figure S4) suggest that the contrast enhancement effects of Fe_3_O_4_-Mal and Fe_3_O_4_-ANG NPs for both subcutaneous and orthotopic glioblastoma models are consistent with the results given in Figure 5. The Prussian blue staining results (Figure 6) show that the blue substance in Fe_3_O_4_-ANG NPs treated groups is more obvious than that of Fe_3_O_4_-Mal NPs groups, further confirming the higher accumulation of iron oxide nanoparticles in tumor site with the help of ANG targeting. The above results demonstrate that the proposed Fe_3_O_4_-ANG NPs have good in vivo targeting ability, enabling the successful detection of glioblastoma through MRI.

## 4. Conclusions

In summary, ultra-small sized Fe_3_O_4_-ANG NPs with excellent colloidal stability and biocompatibility were successfully constructed by covalently coupled ANG onto the particles surface via the PEG ligands. The *r*_1_ of Fe_3_O_4_-ANG NPs was 7.45 mM^−1^s^−1^, which is about 2.2 times higher than that of the Gd-DTPA (3.32 mM^−1^s^−1^). Additionally, the cell-binding test shows that the Fe_3_O_4_-ANG NPs present high binding specificity to U87-L cells. More importantly, the successful in vivo imaging of the subcutaneous and orthotopic glioblastoma model demonstrates the capability of Fe_3_O_4_-ANG NPs to cross the BBB and target the brain tumor, manifesting the possibility for the precise diagnosis of the GBM.

## Data Availability

Data is contained within the article or Supplementary Materials.

## Supplementary Materials

The following are available online at https://www.mdpi.com/article/10.3390/nano11102601/s1, Figure S1: TEM and graphical representation of Fe_3_O_4_-Mal NPs, Figure S2: Standard curve of ANG, Figure S3: Linear regression fitting of the relaxivity of Fe_3_O_4_-Mal and Fe_3_O_4_-ANG NPs for extracting the transverse relaxivity *r*_2_, Figure S4: MRI and tumor signal trends of the subcutaneous glioblastoma and the orthotopic glioblastoma models within 4 h after injection of Fe_3_O_4_-Mal or Fe_3_O_4_-ANG NPs.
