# Supplementary material for: Rational Constructed Ultra-Small Iron Oxide Nanoprobes Manifesting High Performance for T1-Weighted Magnetic Resonance Imaging of Glioblastoma
Source: Nanomaterials (Basel). 2021 Oct 2;11(10):2601. doi: 10.3390/nano11102601 (PMC8540453; doi:10.3390/nano11102601)
Supplement: Supplementary file 1 [file nanomaterials-11-02601-s001.zip › nanomaterials-1390826-supplementary.pdf]

nanomaterials

Supplementary Materials

Rational Constructed Ultra-Small Iron Oxide Nanoprobes Manifesting High Performance for T_1-weighted Magnetic Resonance Imaging of Glioblastoma

Xiangyan Wang [1,2], Lei Chen [2], Jianxian Ge [2], Mohammad Javad Afshari [2], Lei Yang [1,2], Qingqing Miao [2], Ruixue Duan [2], Jiabin Cui [2], Chunyi Liu [2], Jianfeng Zeng [2,*], Jian Zhong [1] and Mingyuan Gao [1,2,*]

[1] College of Food Science & Technology, Shanghai Ocean University, Shanghai 201306, China; wxyixqj@163.com (X.W.); keirayang18@163.com (L.Y.); jzhong@shou.edu.cn (J.Z.)

[2] Center for Molecular Imaging and Nuclear Medicine, State Key Laboratory of Radiation Medicine and Protection, School for Radiological and Interdisciplinary Sciences (RAD-X), Collaborative Innovation Center of Radiological Medicine of Jiangsu Higher Education Institutions, Soochow University, Suzhou 215123, China; chenlei199303@gmail.com (L.C.); jxge@stu.suda.edu.cn (J.G.); moja.afshari@gmail.com (M.J.A.); qqmiao@suda.edu.cn (Q.M.); rxduan@suda.edu.cn (R.D.); jiabin.cui@suda.edu.cn (J.C.); cyliu19@suda.edu.cn (C.L.)

* Correspondence: jfzeng@suda.edu.cn (J.Z.); gaomy@iccas.ac.cn (M.G.)

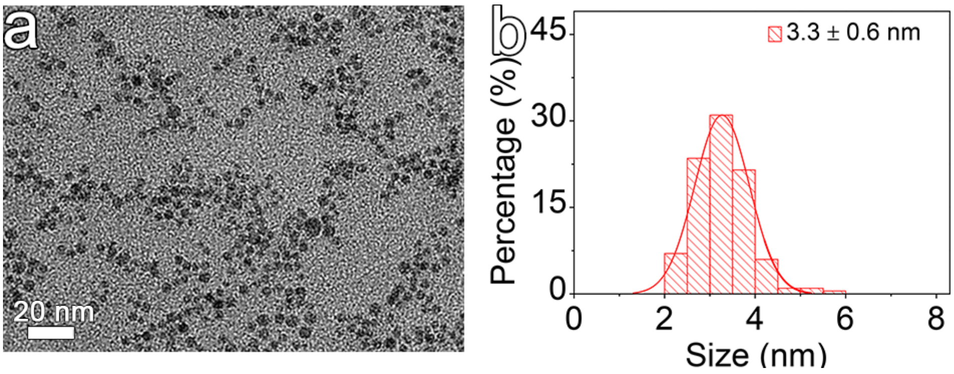

Figure S1. TEM (**a**) and graphical representation (**b**) of Fe_3O_4-Mal NPs.

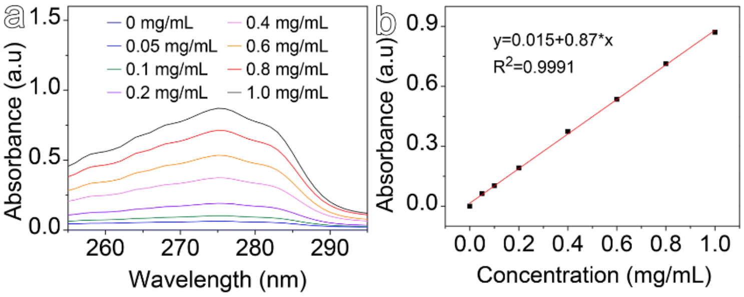

Figure S2. Standard curve of ANG. (**a**) The absorption curve of ANG at different concentrations of 0, 0.05, 0.1, 0.2, 0.4, 0.6, 0.8, 1.0 mg mL^{-1}. (**b**) According to the absorbance of different concentrations of ANG at 275 nm, the standard curve is obtained by fitting a linear equation.

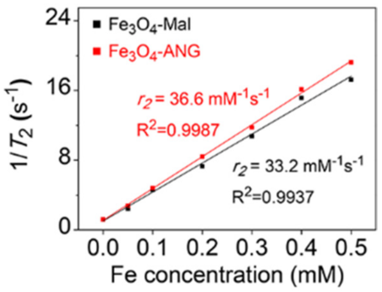

Figure S3. Linear regression fitting of the relaxivity of Fe_3O_4-Mal and Fe_3O_4-ANG NPs for extracting the transverse relaxivity r_2.

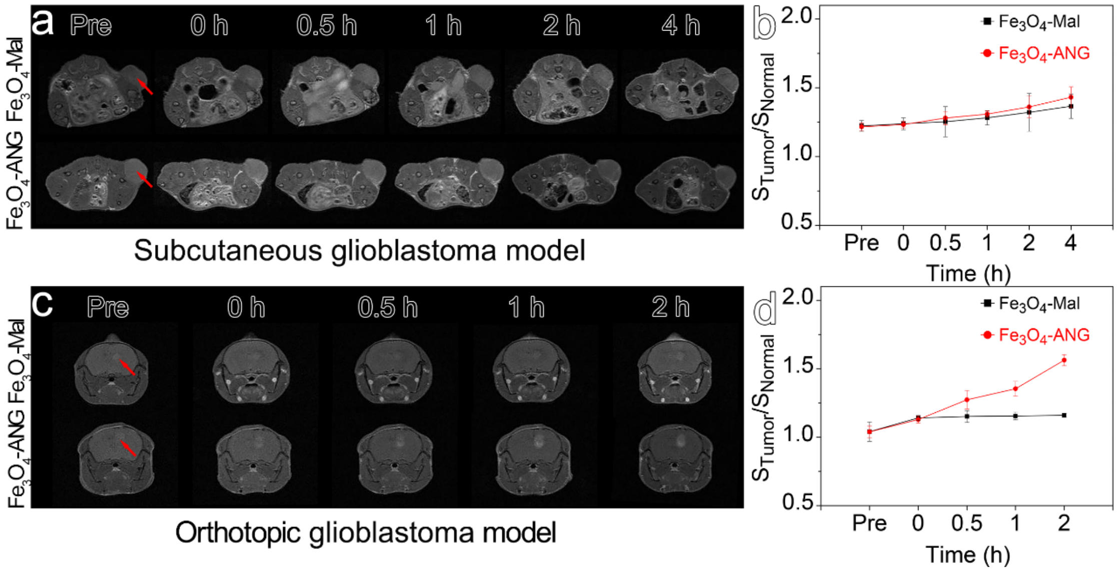

Figure S4. (**a**) MRI of the subcutaneous glioblastoma model within 4 h after injection of Fe_3O_4-Mal or Fe_3O_4-ANG NPs. (**b**) Tumor signal trends for the subcutaneous glioblastoma model after injection of Fe_3O_4-Mal or Fe_3O_4-ANG NPs. (**c**) MRI of the orthotopic glioblastoma model within 2 h after injection of Fe_3O_4-Mal or Fe_3O_4-ANG NPs. (**d**) Tumor signal trends for the orthotopic glioblastoma model after injection of Fe_3O_4-Mal or Fe_3O_4-ANG NPs.
